# No indication for tissue tropism in urogenital and anorectal *Chlamydia trachomatis* infections using high-resolution multilocus sequence typing

**DOI:** 10.1186/1471-2334-14-464

**Published:** 2014-08-26

**Authors:** Bart Versteeg, Martijn S van Rooijen, Maarten F Schim van der Loeff, Henry JC de Vries, Sylvia M Bruisten

**Affiliations:** Public Health Laboratory, Cluster Infectious Diseases, Public Health Service Amsterdam, Amsterdam, The Netherlands; Department of Research, Cluster Infectious Diseases, Public Health Service Amsterdam, Amsterdam, The Netherlands; STI outpatient clinic, Cluster Infectious Diseases, Public Health Service Amsterdam, Amsterdam, The Netherlands; Center for Infections and Immunity Amsterdam (CINIMA), Academic Medical Center, University of Amsterdam, Amsterdam, The Netherlands; Department of Dermatology, Academic Medical Center, University of Amsterdam, Amsterdam, The Netherlands

**Keywords:** *Chlamydia trachomatis*, High-resolution genotyping, Cluster analysis, Men who have sex with men, Sexually transmitted infections

## Abstract

**Background:**

Previous studies showed that *C. trachomatis* strains found in MSM are different from those in heterosexuals. This study investigates whether the differences in strain distribution between MSM and heterosexuals are due to tissue tropism.

**Methods:**

*C. trachomatis* positive samples were collected from MSM (anorectal) and women (anorectal, cervical, vaginal, pharyngeal) visiting the STI outpatient clinic of Amsterdam between 2008 and 2013. All samples were typed using multilocus sequence typing (MLST). Epidemiological data were derived from electronic patient records.

**Results:**

We obtained full MLST data for *C. trachomatis* from 207 MSM and 185 women, all with anorectal infections. Six large clusters were identified of which 3 consisted predominantly of samples from women (89%-100%), whereas the other 3 consisted predominantly of samples from MSM (97%-100%). Furthermore, we obtained full MLST data from 434 samples of 206 women with concurrent infections at multiple anatomical locations. No association was observed between *C. trachomatis* cluster and the anatomical location of infection.

**Conclusion:**

We found no indication for tissue tropism in urogenital, pharyngeal and anorectal *C. trachomatis* infections. Combined with results from previously conducted studies, we hypothesize that MSM and heterosexuals have different distributions of *C. trachomatis* strains due to their separate sexual networks.

**Electronic supplementary material:**

The online version of this article (doi:10.1186/1471-2334-14-464) contains supplementary material, which is available to authorized users.

## Background

*Chlamydia trachomatis* infection is a major public health problem, as it remains the primary cause of bacterial sexually transmitted diseases worldwide [1]. *C. trachomatis* is capable of infecting various cell types and tissues in the human body with a considerable number of infections found in the urogenital tract. Infections also occur in ocular, anorectal and pharyngeal tissue. Most of these infections remain asymptomatic. If not properly treated, these may result in severe complications including epididymitis and pelvic inflammatory disease, leading to infertility in women and possibly also in men [2–4].

*C. trachomatis* is currently divided into 15 main genovars, according to immunotyping of the major outer membrane protein (MOMP) or analysis of the coding *ompA* gene [5]. These 15 genovars can be grouped into ocular genovars A to C, anogenital genovars D to K, and LGV genovars L1 to L3 [6]. Most of the studies using *ompA* typing demonstrated that the majority of infections among heterosexuals involved genovars D, E, and F, while the majority of infections among MSM involved genovars D, G, and J [7–11]. In addition, they also demonstrated that genovar prevalence varied by anatomical site: genovar G is more commonly found in the anorectal tract, whereas other genovars are more common in the urogenital tract [8, 10, 12, 13].

We recently reported that genotyping of *C. trachomatis* using only one molecular target, the *ompA* gene, was far less discriminatory compared to a recently developed high-resolution multilocus sequence typing (MLST) system [14]. This high-resolution MLST system has improved the characterization of strains infecting different populations at risk. Two recent population studies using this MLST method demonstrated distinct transmission networks in MSM and heterosexuals [7, 15]. *C. trachomatis* infections found among heterosexuals belonged to multiple heterogeneous clusters of various sizes, whereas the majority of infections found among MSM belonged to 2 large clusters of strains that circulated exclusively among MSM. One cluster comprised genovar D samples, and the other genovars G and J. However, samples from MSM and heterosexuals included in these population studies were taken from different anatomic sites: samples from MSM were primarily taken from the anorectal tract, whereas samples from heterosexuals were taken from the urogenital tract. The occurrence of these distinct transmission networks between MSM and heterosexuals might therefore also be explained by tissue tropism, causing different *C. trachomatis* sequence types to be preferentially associated with either the urogenital or anorectal tract. This may also explain the previously demonstrated variation in genovar prevalence by anatomical site [8, 10, 12, 13].

Therefore, we investigated whether the differences in MLST identifiable strain distributions between MSM and heterosexuals can be explained by tissue tropism. We assessed: (1) differences in *C. trachomatis* sequence type distributions of anorectal infections between MSM and women, and (2) differences in *C. trachomatis* sequence type distributions by anatomical site in women with concurrent infections at multiple anatomic locations.

## Methods

### Collection of chlamydia-positive samples

For this retrospective analysis we used routinely collected data and samples from women diagnosed with a *C. trachomatis* infection from December 2011 until December 2012 at a visit to the STI outpatient clinic of the Public Health Service of Amsterdam, the Netherlands.

All women were tested for STI according to standard procedures, as described previously [7, 16]. In brief, swabs were taken either from the vagina, cervix, urethra, (all considered urogenital infections), rectum, or pharynx, depending on sexual techniques, risk behaviour, clinical signs, being notified of an STI, and symptoms associated with chlamydial infections and other STIs. All collected swabs were tested for the presence of *C. trachomatis* RNA using the Aptima Combo 2 assay (Hologic/Gen-Probe, San Diego, CA) at the Public Health Laboratory, Amsterdam. Positive *C. trachomatis* samples were stored at −20°C. This analysis was restricted to women who were diagnosed with either concurrent *C. trachomatis* infections at multiple anatomic locations, or with solitary anorectal infections. Additional demographic and sexual risk behaviour data were obtained from the electronic patient records from the STI clinic, which contained data on gender, age, number of sexual partners in the preceding 6 months, sexual techniques, being notified by a sexual partner, STI related symptoms, HIV status, and having received money for sex in the preceding 6 months.

For comparison of anorectal infections among MSM and women, we selected data from MSM with anorectal *C. trachomatis* infections from a previous study [7]. These were MSM visiting the same STI clinic between July 2008 and August 2009, who reported having had receptive anal sex with a man in the preceding 6 months. Full MLST data and additional demographic and sexual risk behaviour data (from electronic patient records) were available for all selected MSM [7].

The Medical Ethical Committee of Academic Medical Center of the University of Amsterdam, The Netherlands approved this study.

### DNA amplification

DNA from all included clinical samples was extracted by isopropanol precipitation and tested for the presence of chlamydial DNA using an in-house *pmpH* LGV qPCR [11, 17]. For isolates that tested negative, DNA was re-extracted from the original samples and retested. All samples that repeatedly tested negative were excluded. For all isolates that tested positive, samples were aggregated in two categories: (1) samples with a cycling threshold lower than or equal to 35, and (2) isolates with a cycling threshold higher than 35. The latter were usually unsuitable for further typing.

### Nested PCR and sequencing of MLST regions

DNA isolates were amplified by a nested PCR for the regions *ompA*, CT046 (*hctB*), CT058, CT144, CT172, and CT682 (*pbpB*) as described previously [7, 14]. The inner PCR for isolates with a qPCR cycling threshold lower than or equal to 35 were performed with M13-tagged primers, similar to the standard inner primers. The inner PCR for isolates with a qPCR cycling threshold higher than 35 were performed with standard inner primers. The M13-tagged amplified DNA samples were sent to the sequence facility of the Academical Medical Center of Amsterdam for further processing. The non M13-tagged amplified DNA was processed at the Public Health Laboratory as described previously [14].

### MLST data analysis

The obtained sequences were assembled and trimmed, using BioNumerics 7 (Applied Maths, Sint-Martens-Latem, Belgium). The sequences were checked against the *C. trachomatis* MLST database (http://mlstdb.bmc.uu.se). Only samples in which all six loci were successfully amplified, sequenced, and identified obtained a full MLST profile (Sequence type, ST) and were included in further analysis. Incomplete and low quality samples were re-amplified and re-sequenced at the Public Health Laboratory. Minimum spanning trees were generated with BioNumerics 7 using the MLST profiles. A cluster was defined as a group of STs differing by not more than one locus from another ST within that group. Clusters containing 10 or more samples were defined as large clusters. To investigate specific characteristics of small clusters (n < 10) and singletons, we combined these samples into a residual group.

### Statistics

*C. trachomatis* infections were aggregated into cervical, urethral, vaginal, anorectal or pharyngeal infections, based on the anatomical location of sampling. Paired samples of women with concurrent infections at multiple anatomic locations were aggregated into concordant or discordant infections based on sequence type variation between the samples. Paired samples with sequence types that belonged to the same cluster were considered concordant infections, whereas paired samples with sequence types that belonged to different clusters were considered discordant infections. All women were regarded as heterosexuals, as female-to-female transmission of *C. trachomatis* is very rare [18].

Differences between groups and clusters were tested using the Pearson *χ*^2^ test for categorical data. Fisher exact test was used when an expected cell count was <1. For continuous variables Mann–Whitney U tests and Kruskall-Wallis tests were used. A *P* value <0.05 was considered statistically significant. Generalized estimating equations (GEE) was used to account for possible correlations among multiple samples of women with discordant infections. Analyses were performed with SPSS package version 21.0 (SPSS Inc., Chicago, IL, USA).

## Results

### Study population and specimens

**Figure 1:**
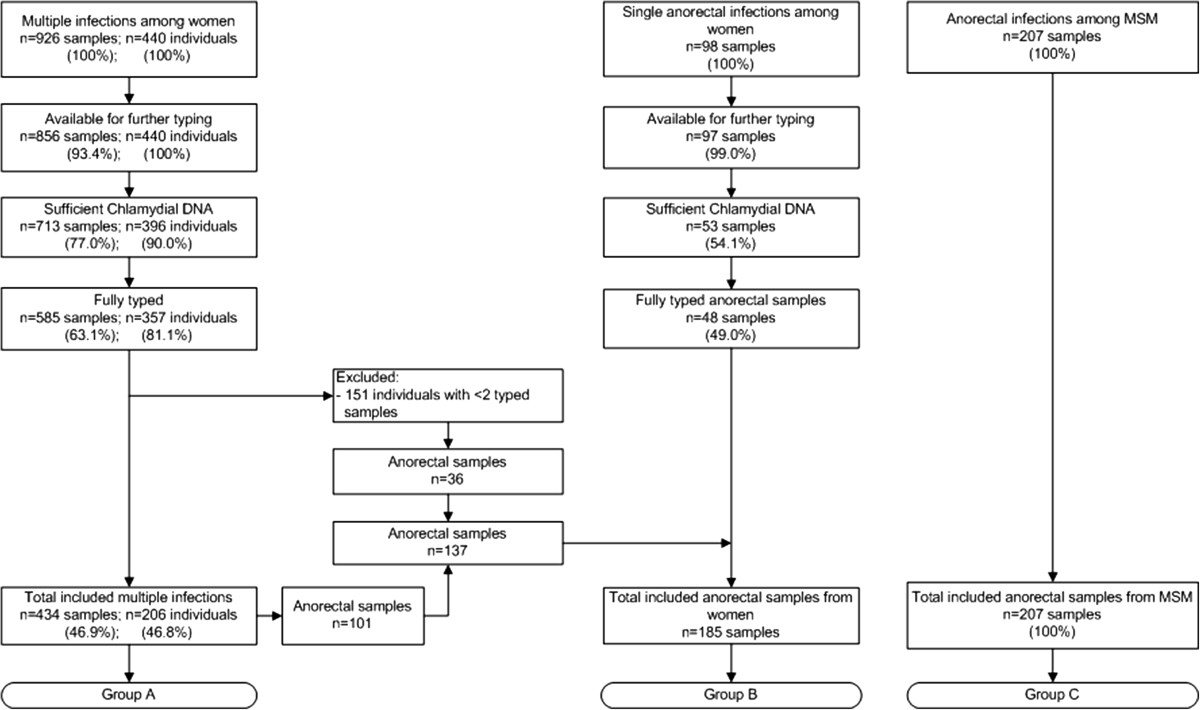
**Flowchart indicating the number of samples and individuals included and excluded from the study population.** Amsterdam 2008–2012. The MSM samples were previously described [7].

From December 2011 to December 2012, a total of 17,343 women visited the STI outpatient clinic of the Public Health Service of Amsterdam, of whom 7,143 were tested for chlamydia on multiple anatomical locations during the same consultation. Of those, 440 (6.2%) tested positive for *C. trachomatis* infections on multiple anatomic locations, resulting in a total of 926 chlamydia positive samples (Figure 1). Of these, 856 (92.4%) were available for typing analysis. In 713 samples (83.3%), sufficient chlamydial DNA could be demonstrated using qPCR, and for 585 of these samples (63.1%) derived from 357 women, full MLST profiles could be obtained. We excluded 151 women with successfully typed samples from only one location (Figure 1; group A). The remaining 206 women provided 434 samples comprising 149 (34.3%) cervical samples, 116 (26.7%) urethral samples, 51 (11.8%) vaginal samples, 101 (23.3%) anorectal samples, and 17 (3.9%) pharyngeal samples.

During the same period, 98 women were diagnosed with a solitary anorectal *C. trachomatis* infection. Of these, 97 (99.0%) samples were available for further testing. In 53 of these samples (54.1%) sufficient chlamydial DNA could be demonstrated using qPCR. For 48 of them (90.6%), full MLST profiles could be obtained. In addition to these 48 samples, we had 137 anorectal samples with full MLST profiles derived from group A (Figure 1). Together these 185 anorectal samples constituted group B (Figure 1). Overall, no significant differences were observed between women included and excluded from the study for age, ethnicity, number of sexual partners in the previous 6 months, vaginal intercourse, receptive anal intercourse, active oral intercourse, being notified of an STI by a sexual partner, STI related complaints, HIV status and having received money for sex in the previous 6 months. For comparison, 207 anorectal samples from MSM with a full MLST profile were included from a previous study [7] (Figure 1; group C).

### Comparison of MSM and women with anorectal infections

**Figure 2:**
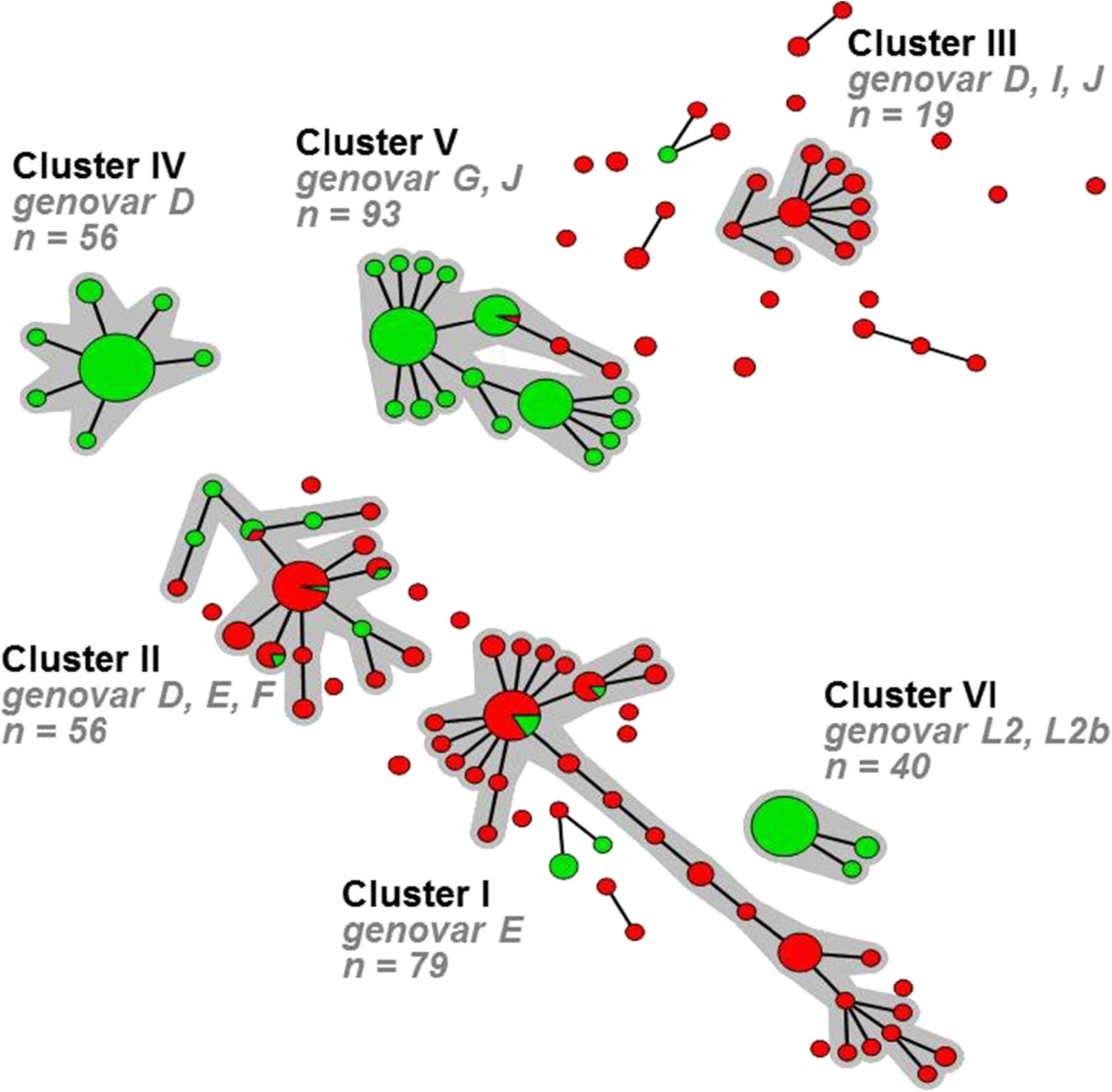
**Minimum spanning tree showing the MLST pattern of 392 Chlamydia trachomatis positive anorectal samples from MSM and women.** Each circle represents one ST. Size of the circles is proportional to the number of identical ST profiles. Bold lines connect types that differ for one single locus. Halos indicate the distinct clusters. The color coding is: green, men who have sex with men (n = 207); red, heterosexual women (n = 185).

Data of 185 anorectal samples from women (group B; Figure 1) and 207 anorectal samples from MSM (group C, Figure 1) were available for analysis. Almost all demographic and sexual risk behaviour characteristics differed significantly between MSM and women (Table 1). The median age of all women with a fully typed anorectal sample was 23 years (IQR 20–26 years) and their median number of partners during the last 6 months was 3 (IQR 1–5). In comparison, MSM were significantly older (*P* <0.001) with a median age of 38 years (IQR 31–45 years) and reported more sexual partners during the last 6 months, with a median of 10 partners (IQR 4–20; *P* < 0.001). In addition, MSM were more frequently HIV positive (*P* < 0.001), but fewer MSM reported having received money for sex in the previous 6 months (*P* < 0.001; Table 1).Using the complete MLST profiles of all 392 anorectal samples of MSM and women, 119 STs could be identified. Of these STs 38 had multiple representatives (2 to 47 isolates) while 81 were found in only a single isolate (singletons). Using these STs, a minimum spanning tree was generated, in which 6 large clusters could be identified (Figure 2). These clusters ranged from 19 to 93 samples comprising 87.5% of all samples. The remaining 49 samples from MSM and women had more than one locus difference compared to other samples that were included in the large clusters, and therefore constituted the residual group. These remaining samples were distributed over 23 singletons and 6 small clusters, ranging from 2 to 7 samples. The minimum spanning tree shows a clear distinction between samples from MSM and women. Of the 6 large clusters, 3 consisted predominantly of samples from women (83.9% to 100%) whereas the other 3 large clusters consisted predominantly of samples from MSM (96.8% to 100%). Outside the identified large clusters, 7 samples were from MSM and 42 samples were from women. Overall, the samples from women showed more genetic diversity than those from MSM.

**Table 1:** **Demographic and sexual risk behavior characteristics of women and men who have sex with men (MSM) with anorectal** ***Chlamydia trachomatis*** **infections**

Variable	Total (N = 392)			Women (N = 185)			MSM (N = 207)			
	*	n	(%)	*	n	(%)	*	n	(%)	*P*
**Demographics**										
Age in years (continuous)	383			185			198			**<0.001**
Median (IQR)		28	(22–39)		23	(20–26)		38	(31–45)	
Age in years (categorical)	383			185			198			**<0.001**
≤15		1	(0.3%)		1	(0.5%)		0	(0.0%)	
15-19		25	(6.5%)		25	(13.5%)		0	(0.0%)	
20-24		107	(27.9%)		95	(51.4%)		12	(6.1%)	
25-29		72	(18.8%)		41	(22.2%)		31	(15.7%)	
30-39		84	(21.9%)		15	(8.1%)		69	(34.8%)	
≥40		94	(24.5%)		8	(4.3%)		86	(43.4%)	
**Sexual behavior**										
Number of partners in preceding 6 months (continuous)	286			185			188			**<0.001**
Median (IQR)		4	(2–10)		3	(1–5)		10	(4–20)	
Receptive anal intercourse	381			183			198			**<0.001**
None^a^		31	(8.1%)		22	(12.0%)		1	(0.5%)	
Safe		70	(18.4%)		16	(8.7%)		54	(27.3%)	
Unsafe^b^		280	(73.5%)		145	(79.2%)		143	(72.2%)	
Notified of STI by a sexual partner	383			185			198			0.961
No		306	(79.9%)		148	(80.0%)		158	(79.8%)	
Yes		77	(20.1%)		37	(20.0%)		40	(20.2%)	
STI related symptoms	383			185			198			**<0.001**
No		233	(60.8%)		132	(71.4%)		101	(51.0%)	
Yes		150	(39.2%)		53	(28.6%)		97	(49.0%)	
HIV status	392			185			207			**<0.001**
Unknown		19	(4.8%)		3	(1.6%)		16	(7.7%)	
Negative		278	(70.9%)		182	(98.4%)		96	(46.4%)	
Positive		95	(24.2%)		0	(0.0%)		95	(45.9%)	
Received money for sex in preceding 6 months	383			185			198			**<0.001**
No		354	(92.4%)		158	(85.4%)		196	(99.0%)	
Yes		29	(7.6%)		27	(14.6%)		2	(1.0%)	

*Abbreviations:* *MSM* men who have sex with men, *STI* sexual transmitted infection, *HIV* human immunodeficiency virus, *IQR* interquartile range, *P P*-value.

Significant *P*-values in **bold**.

*Number of individuals with available data.

^a^Of all women reporting no receptive anal intercourse, 14 were prostitutes and were also tested for rectal *C. trachomatis* infections following routine guidelines, 5 women were accidently tested, 3 women were tested for reporting anal discharge, and 1 women was tested due to notification by a sexual partner.

^b^Eight women reported receptive anal intercourse but condom use was unknown. These were all included as having unsafe receptive anal intercourse, as this would be the most likely explanation for infection.

The clusters dominated by women consisted of genovars D, E, F, I, and J, with genovars D, E, and F being most prominent. In comparison, the clusters dominated by MSM consisted of genovars D, G, J, L2, and L2b, with genovars D, G, J, and L2b being most prominent. The residual group, consisting of the remaining small clusters and singletons, included a wide variety of genovars, but genovars D and G were most common (Table 2).

**Table 2:** **Demographic and sexual risk behavior characteristics of women and MSM with anorectal infections by** ***Chlamydia trachomatis*** **MLST cluster**

Variable	Cluster I		Cluster II		Cluster III		Cluster IV		Cluster V		Cluster VI		Residual		*P*_I-III+R_	*P*_IV-VI_	*P*_IV+V_	*P*_total_
	n = 79		n = 56		n = 19		n = 93		n = 56		n = 40		n = 49					
*ompA* Genovar															-	-	-	-
B	0	(0.0%)	0	(0.0%)	0	(0.0%)	0	(0.0%)	0	(0.0%)	0	(0.0%)	1	(2.0%)				
D	0	(0.0%)	9	(16.1%)	1	(5.3%)	0	(0.0%)	56	(100.0%)	0	(0.0%)	12	(24.5%)				
E	79	(100.0%)	4	(7.1%)	0	(0.0%)	0	(0.0%)	0	(0.0%)	0	(0.0%)	8	(16.3%)				
F	0	(0.0%)	43	(76.8%)	0	(0.0%)	0	(0.0%)	0	(0.0%)	0	(0.0%)	2	(4.1%)				
G	0	(0.0%)	0	(0.0%)	0	(0.0%)	64	(68.8%)	0	(0.0%)	0	(0.0%)	10	(20.4%)				
H	0	(0.0%)	0	(0.0%)	0	(0.0%)	0	(0.0%)	0	(0.0%)	0	(0.0%)	4	(8.2%)				
I	0	(0.0%)	0	(0.0%)	15	(78.9%)	0	(0.0%)	0	(0.0%)	0	(0.0%)	3	(6.1%)				
J	0	(0.0%)	0	(0.0%)	3	(15.8%)	29	(31.2%)	0	(0.0%)	0	(0.0%)	6	(12.2%)				
K	0	(0.0%)	0	(0.0%)	0	(0.0%)	0	(0.0%)	0	(0.0%)	0	(0.0%)	3	(6.1%)				
L2	0	(0.0%)	0	(0.0%)	0	(0.0%)	0	(0.0%)	0	(0.0%)	3	(7.5%)	0	(0.0%)				
L2b	0	(0.0%)	0	(0.0%)	0	(0.0%)	0	(0.0%)	0	(0.0%)	37	(92.5%)	0	(0.0%)				
**Demographics**																		
Gender															0.095	0.207	0.175	**<0.001**
Male	5	(6.3%)	9	(16.1%)	0	(0.0%)	90	(96.8%)	56	(100.0%)	40	(100.0%)	7	(14.3%)				
Female	74	(93.7%)	47	(83.9%)	19	(100.0%)	3	(3.2%)	0	(0.0%)	0	(0.0%)	42	(85.7%)				
Age in years (continuous)^a^															0.438	0.112	0.089	**<0.001**
Median (IQR)	23	(21–27)	23	(20–28)	22	(21–24)	37	(29–45)	38	(29–45)	43	(35–46)	25	(20–29)				
Age in years (categorical)^a^															0.656	0.359	0.951	**<0.001**
<15	0	(0.0%)	1	(1.8%)	0	(0.0%)	0	(0.0%)	0	(0.0%)	0	(0.0%)	0	(0.0%)				
15-19	10	(12.7%)	7	(12.7%)	3	(0.0%)	0	(0.0%)	0	(0.0%)	0	(0.0%)	5	(10.2%)				
20-24	40	(50.6%)	25	(45.5%)	12	(0.0%)	6	(6.9%)	5	(9.1%)	0	(0.0%)	19	(38.8%)				
25-29	14	(17.7%)	13	(23.6%)	2	(0.0%)	16	(18.4%)	9	(16.4%)	3	(7.7%)	15	(30.6%)				
30-39	12	(15.2%)	6	(10.9%)	1	(0.0%)	27	(31.0%)	18	(32.7%)	15	(38.5%)	5	(10.2%)				
≥40	3	(3.8%)	3	(5.5%)	1	(0.0%)	38	(43.7%)	23	(41.8%)	21	(53.8%)	5	(10.2%)				
**Sexual behavior**																		
Number of partners in preceding 6 months (continuous)^b^															0.396	**0.014**	0.463	**<0.001**
Median (IQR)	3	(2–6)	3	(2–6)	2	(1–3)	5	(3–20)	5	(3–10)	10	(10–20)	3	(1–6)				
Receptive anal intercourse^c^															0.863	0.056	0.524	**<0.001**
None	9	(16.7%)	4	(13.0%)	1	(10.5%)	2	(2.3%)	0	(0.0%)	0	(0.0%)	7	(14.3%)				
Safe	7	(9.0%)	7	(13.0%)	2	(10.5%)	27	(31.0%)	17	(30.9%)	4	(10.3%)	6	(12.2%)				
Unsafe^d^	62	(74.4%)	43	(74.1%)	16	(78.9%)	58	(66.7%)	38	(69.1%)	35	(89.7%)	36	(73.5%)				
Notified of STI by a sexual partner^a^															0.301	0.740	0.750	0.556
No	69	(87.3%)	41	(74.5%)	15	(78.9%)	66	(75.9%)	43	(78.2%)	32	(82.1%)	40	(81.6%)				
Yes	10	(12.7%)	14	(25.5%)	4	(21.1%)	21	(24.1%)	12	(21.8%)	7	(17.9%)	9	(18.4%)				
STI related symptoms^a^															0.778	**0.007**	0.548	**0.014**
No	55	(69.6%)	34	(61.8%)	13	(68.4%)	55	(63.2%)	32	(58.2%)	13	(33.3%)	31	(63.3%)				
Yes	24	(30.4%)	21	(38.2%)	6	(31.6%)	32	(36.8%)	23	(41.8%)	26	(66.7%)	18	(36.7%)				
HIV status															0.627	**<0.001**	0.556	**<0.001**
Unknown	0	(0.0%)	2	(3.6%)	1	(5.3%)	9	(9.7%)	3	(5.4%)	3	(7.5%)	1	(2.0%)				
Negative	75	(94.9%)	52	(92.9%)	18	(94.7%)	53	(57.0%)	31	(55.4%)	3	(7.5%)	46	(93.9%)				
Positive	4	(5.1%)	2	(3.6%)	0	(0.0%)	31	(33.3%)	22	(39.3%)	34	(85.0%)	2	(4.1%)				
Received money for sex in preceding 6 months^a^															0.822	0.581	0.425	**<0.001**
No	65	(82.3%)	50	(90.9%)	17	(89.5%)	86	(98.9%)	55	(100.0%)	39	(100.0%)	42	(85.7%)				
Yes	5	(6.3%)	5	(9.1%)	2	(10.5%)	1	(1.1%)	0	(0.0%)	0	(0.0%)	7	(14.3%)				

*Abbreviations:* *MSM* men who have sex with men, *STI* sexual transmitted infection, *HIV* human immunodeficiency virus, *IQR* interquartile range, *P* P-value.

P_total_ represents the p-value for the analysis over all clusters and the residual group; P_I-III+R_ over clusters I to III and the residual group consisting predominantly of women; P_IV-VI_ over clusters VI tot VI consisting predominantly of MSM including the LGV cluster. P_IV-V_ over clusters IV and V consisting predominantly of MSM excluding the LGV cluster.

Significant *P*-values in **bold**.

^a^Data is missing for 1 cluster II infection, 6 cluster IV infections, 1 cluster V infection, and 1 cluster VI infection.

^b^Data is missing for 3 cluster I infections, 4 cluster II infections, 45 cluster IV infections, 16 cluster VI infections, and 4 residual infections.

^c^Data is missing for 1 cluster I infection, 2 cluster II infections, 6 cluster IV infections 1 cluster V infection, and 1 cluster VI infection.

^d^Eight women reported receptive anal intercourse but condom use was unknown. These were all included as having unsafe receptive anal intercourse, as this would be the most likely explanation for infection.

### Cluster analysis of MSM and women with anorectal infections

We observed significant differences for gender, age, number of partners in the preceding 6 months, receptive anal intercourse, STI related symptoms, HIV status and having received money for sex in the preceding 6 months between all clusters. However, no significant differences could be observed when comparing only the residual group and the clusters dominated by women (cluster I-III; P_I-III+R_, Table 2). These findings are fully in line with previous findings by our group in which distinct circulating *C. trachomatis* strains were found among MSM and heterosexuals, as was discussed elsewhere [7].

Comparing the MSM dominated clusters (cluster IV-VI, Table 2), significant differences were observed in the number of sexual partners in the preceding 6 months (*P =* 0.014; *P*_IV-VI_ Table 2). MSM in the LGV cluster reported more sexual partners (median of 10 (IQR, 10–20)) compared to MSM in the other MSM dominated clusters (median of 5 (IQR, 3–20)). Significant differences were also observed for STI related complaints (*P =* 0.007; *P*_IV-VI_ Table 2). A larger proportion of MSM in the LGV cluster reported STI related complaints (66.7%) compared to MSM in the other MSM dominated clusters (36.8 to 41.8%). Finally, significant differences were observed for HIV status (*P* < 0.001; *P*_IV-VI_ Table 2) A larger proportion of MSM in the LGV cluster were HIV positive (85.0%) compared to MSM in the other MSM dominated clusters (33.3 to 39.3%). These differences were therefore mainly due to the LGV cluster that is known to prevail among a core group of high risk MSM [7, 19, 20]. When we excluded the LGV cluster (cluster VI) from the analysis, no significant differences were observed between the remaining two MSM clusters, as was shown previously by our group [7].

### Comparison of women with concurrent infections at multiple anatomic locations

**Figure 3:**
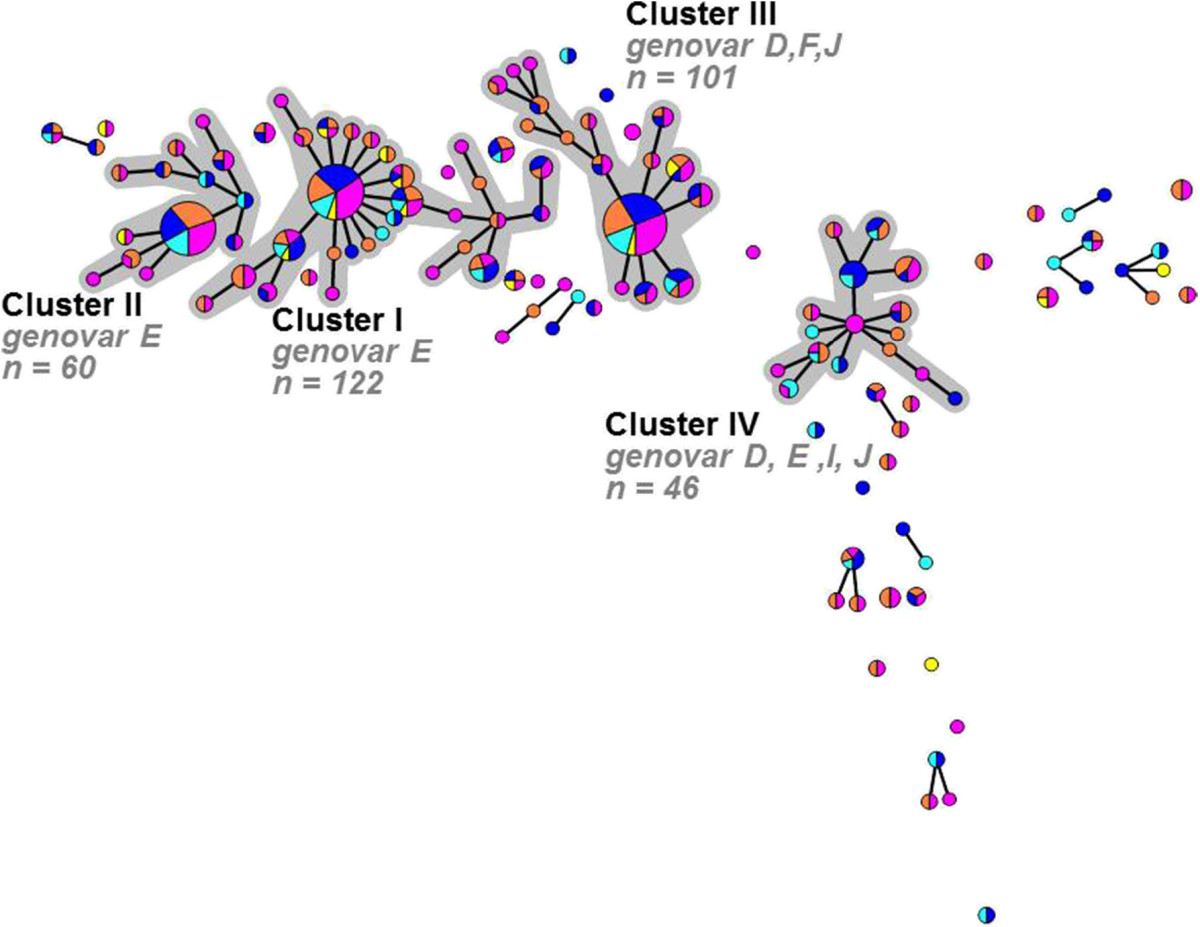
**Minimum spanning tree showing the MLST pattern of 434 Chlamydia trachomatis positive samples from 206 women with concurrent infections at multiple anatomic locations.** Each circle represents one MLST type. Size of the circles is proportional to the number of identical STs. Bold lines connect types that differ for one single locus. Halos indicate the distinct clusters. Colors indicate the anatomic location of sampling; pink: cervical samples (n = 149), cyan: urethral samples (n = 116), orange: vaginal samples (n = 51), blue: anorectal samples (n = 101), and yellow: pharyngeal samples (n = 17).

The median age of the 206 women with concurrent infections at multiple anatomic locations (group A, Figure 1) was 22 years (IQR 16–28 years) and their median number of partners during the last 6 months was 3 (IQR 1–6) (Table 3). Using the complete MLST profile of all 434 samples of these 206 women, 126 unique STs could be identified of which 51 were novel to the *C. trachomatis* MLST database (http://mlstdb.bmc.uu.se). Novel STs were numbered in order of identification and were found in 88 (20.1%) of 434 samples. Of all identified STs 79 had multiple representatives (2 to 50 isolates) while 47 were found in only a single isolate (singletons). Using the STs of all of these samples, a minimum spanning tree was generated, in which 4 large clusters could be identified (Figure 3). These clusters ranged from 46 to 123 samples comprising 75.3% of all samples. The remaining 108 samples were distributed over 47 singletons and 10 small clusters, ranging from 2 to 9 samples.

**Table 3:** **Demographic and sexual risk behavior characteristics of women with concurrent concordant- and discordant multiple C** ***hlamydia trachomatis*** **infections**

Variable	Total (N = 206)			Concordant (N = 179)			Discordant (N = 27)			
	*	n	(%)	*	n	(%)	*	n	(%)	*P*
**Demographics**										
Age in years (continuous)	206			179			27			0.321
Median (IQR)		22	(20–26)		22	(20–26)		21	(19–26)	
Age in years (categorical)	206			179			27			0.213
<15		0	(0.0%)		0	(0.0%)		0	(0.0%)	
15-19		38	(18.4%)		30	(16.8%)		8	(29.6%)	
20-24		105	(51.0%)		95	(53.1%)		10	(37.0%)	
25-29		43	(20.9%)		35	(19.6%)		8	(29.6%)	
30-39		13	(6.3%)		12	(6.7%)		1	(3.7%)	
≥40		7	(3.4%)		7	(3.9%)		0	(0.0%)	
Ethnicity	206			179			27			0.327
Dutch		97	(47.1%)		84	(46.9%)		13	(48.1%)	
Surinamese		38	(18.4%)		30	(16.8%)		8	(29.6%)	
Eastern European		22	(10.7%)		20	(11.2%)		2	(7.4%)	
Other		49	(23.8%)		45	(25.1%)		4	(14.8%)	
**Sexual behavior**										
Number of partners in preceding 6 months (continuous)	206			179			27			0.545
Median (IQR)		3	(2–5)		3	(2–5)		3	(1–6)	
Vaginal intercourse	203			177			26			0.831
Safe		21	(10.3%)		18	(10.2%)		3	(11.5%)	
Unsafe		182	(89.7%)		159	(89.8%)		23	(88.5%)	
Receptive anal intercourse	205			179			26			0.797
None		110	(53.7%)		97	(54.2%)		13	(50.0%)	
Safe		17	(8.3%)		14	(7.8%)		3	(11.5%)	
Unsafe		78	(38.0%)		68	(38.0%)		10	(38.5%)	
Active oral intercourse	203			177			26			0.969
None		28	(13.8%)		24	(13.6%)		4	(15.4%)	
Safe		8	(3.9%)		7	(4.0%)		1	(3.8%)	
Unsafe		167	(82.3%)		146	(82.5%)		21	(80.8%)	
Notified of STI by a sexual partner	206			179			27			0.435
No		143	(69.4%)		126	(70.4%)		17	(63.0%)	
Yes		63	(30.6%)		53	(29.6%)		10	(37.0%)	
STI related symptoms	206			179			27			0.209
No		87	(42.2%)		70	(39.1%)		17	(63.0%)	
Yes		122	(59.2%)		109	(60.9%)		13	(48.1%)	
HIV status	206			179			27			0.679
Unknown		4	(1.9%)		4	(2.2%)		0	(0.0%)	
Negative		201	(97.6%)		174	(97.2%)		27	(100.0%)	
Positive		1	(0.5%)		1	(0.6%)		0	(0.0%)	
Received money for sex in preceding 6 months	206			179			27			0.263
Yes		175	(85.0%)		154	(86.0%)		21	(77.8%)	
No		31	(15.0%)		25	(14.0%)		6	(22.2%)	

*Abbreviations:* *STI* sexual transmitted infection, *HIV* human immunodeficiency virus, *IQR* interquartile range, *P P*-value.

Significant *P*-values in **bold**.

*Number of individuals with available data.

The minimum spanning tree shows a heterogeneous distribution of STs found per anatomic location (Figure 3). The 4 large clusters contained genovars D, E, F, I, and J, with genovars D, E, and F being most prominent. The residual group containing the remaining small clusters and singletons included all genovars with a majority of genovars D, E, and G. Overall, no significant differences could be observed when comparing the proportions of cervical, urethral, vaginal, anorectal or pharyngeal infections between all clusters and the residual group (Table 4).

**Table 4:** **Characteristics of** ***Chlamydia trachomatis*** **infections among women with concurrent infections at multiple anatomic locations, by** ***Chlamydia trachomatis*** **MLST cluster**

Variable	Cluster I		Cluster II		Cluster III		Cluster IV		Residual		***P***
	n = 121		n = 60		n = 99		n = 46		n = 108		
*ompA* genovar											-
B	0	(0.0%)	0	(0.0%)	0	(0.0%)	0	(0.0%)	4	(3.7%)	
D	0	(0.0%)	0	(0.0%)	19	(19.2%)	2	(4.3%)	18	(16.7%)	
E	121	(100.0%)	60	(100.0%)	0	(0.0%)	1	(2.2%)	21	(19.4%)	
F	0	(0.0%)	0	(0.0%)	74	(74.7%)	0	(0.0%)	10	(9.3%)	
G	0	(0.0%)	0	(0.0%)	0	(0.0%)	0	(0.0%)	24	(22.2%)	
H	0	(0.0%)	0	(0.0%)	0	(0.0%)	0	(0.0%)	9	(8.3%)	
I	0	(0.0%)	0	(0.0%)	0	(0.0%)	34	(73.9%)	3	(2.8%)	
J	0	(0.0%)	0	(0.0%)	6	(6.1%)	9	(19.6%)	12	(11.1%)	
K	0	(0.0%)	0	(0.0%)	0	(0.0%)	0	(0.0%)	7	(6.5%)	
Anatomic location of infection											0.968
Cervical	40	(33.1%)	21	(35.0%)	37	(37.4%)	13	(28.3%)	38	(35.2%)	
Urethral	33	(27.3%)	17	(28.3%)	25	(25.3%)	13	(28.3%)	28	(25.9%)	
Vaginal	13	(10.7%)	8	(13.3%)	9	(9.1%)	8	(17.4%)	13	(12.0%)	
Anorectal	28	(23.1%)	13	(21.7%)	24	(24.2%)	12	(26.1%)	24	(22.2%)	
Pharyngeal	7	(5.8%)	1	(1.7%)	4	(4.0%)	0	(0.0%)	5	(4.6%)	
Discordant multiple infections^a^											0.330^*^
No	110	(90.9%)	51	(85.0%)	87	(87.9%)	40	(87.0%)	88	(81.5%)	
Yes	11	(9.1%)	9	(15.0%)	12	(12.1%)	6	(13.0%)	20	(18.5%)	

*Abbreviation:* *P* P-value.

^*^GEE is used.

^a^Discordant multiple infections defined as: having ≥ 2 paired samples with sequence types that belonged to different clusters.

### Discordant infections among women with concurrent infections at multiple anatomic locations

**Figure 4:**
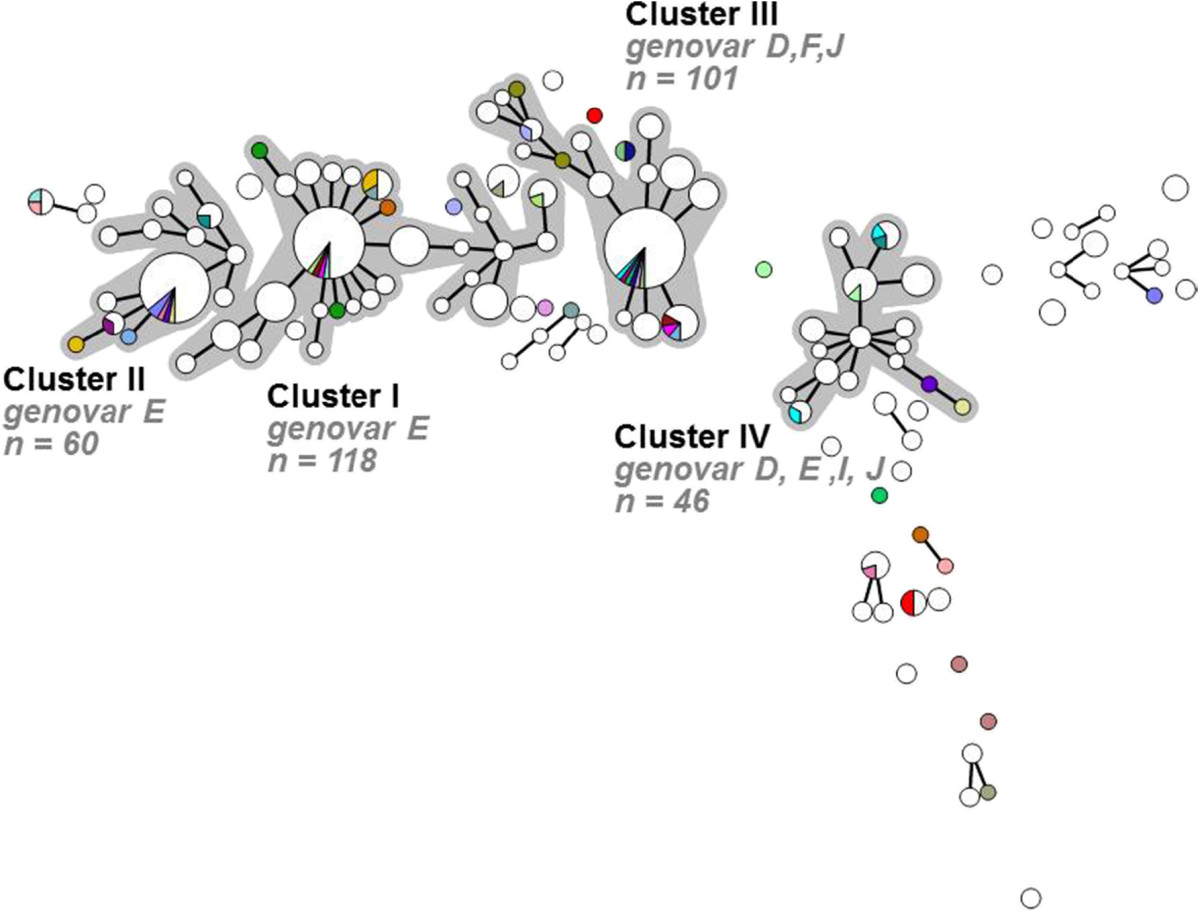
**Minimum spanning tree showing the MLST pattern of 434 Chlamydia trachomatis positive samples from 206 women with concurrent infections at multiple anatomic locations.** Each circle represents one MLST type. Size of the circles is proportional to the number of identical STs. Bold lines connect types that differ for one single locus. Halos indicate the distinct clusters. White indicates concordant samples (maximum 1 locus difference in MLST profile between infections) within one woman. Colors indicate discordant samples, with a unique color for each of the 27 women. Discordant is more than 1 locus difference in MLST profile between infections.

Analysis of STs found among all samples from 206 women with concurrent infections at multiple anatomic locations identified 376 samples from 179 women (86.9%; 95% CI [81.8-91.0%]), in whom all concurrent infections were caused by the same (concordant) chlamydia strains belonging to the same clusters. For 264 samples from 153 (74.3%; 95% CI [68.0-79.9%]) of these women, concordant strains had an identical ST, and for 54 samples from 26 (12.6%; 95% CI [8.6-17.6%]) women the ST differed at 1 locus between concurrent samples. The remaining 58 samples belonged to 27 women (13.1%; 95% CI [9.0-18.2%]) who had concurrent infections caused by discordant *C. trachomatis* strains, belonging to different clusters. Of those, 6 women had concurrent samples that differed at 2 loci and 21women had concurrent samples that differed at 3 or more loci. Statistical analysis of demographic and sexual risk behaviour characteristics revealed no significant differences between women with concordant and discordant infections (Table 3). In addition, no significant differences could be observed when comparing the proportion of concordant and discordant infections between all clusters and the residual group (Figure 4; Table 4).

## Discussion

In this study, we observed that anorectal *C. trachomatis* infections in women were caused by different strains than anorectal *C. trachomatis* infections in MSM. We eliminated the discrepancy in anatomical sample site and still observed largely distinct *C. trachomatis* strains infecting MSM and women. We found that *C. trachomatis* infections circulating among women belonged to 3 heterogeneous clusters of various sizes, whereas the majority of infections found among MSM belonged to 3 homogeneous clusters.

We did not include *C. trachomatis* strains from male urethra since this is a common factor in the transmission among both MSM and heterosexual women. As a result this would not add discriminative data to a possible tissue related role in the distribution of strains between the 2 populations. Previous studies hypothesized that pathogen-related factors such as tissue tropism could explain the distinct distribution of *C. trachomatis* strains circulating among MSM and heterosexual populations, but lacked data to test this hypothesis [7, 15]. We now tested this hypothesis and compared identical anatomical sites between MSM and women, but observed hardly any overlap of strains circulating among MSM and women suggesting that these distinct distributions of *C. trachomatis* strains is not caused by tissue tropism. Therefore, we hypothesize that the distinct distributions of *C. trachomatis* strains among MSM and women is caused by network factors, and that limited transmission of *C. trachomatis* strains occurs between MSM and heterosexual networks. Moreover, sexual behaviour may also influence the distinct distributions of *C. trachomatis* strains among MSM and women as we observed differences in demographic and sexual risk behaviour characteristics between MSM and women: MSM were significantly older and had had more sexual partners in the previous 6 months. We observed no differences in demographic, or sexual behaviour characteristics between clusters consisting of samples derived from women.

We focused on tissue tropism in cervical, urethral, vaginal, anorectal and pharyngeal infections that are primarily caused by the anogenital genovars D to K, and the LGV genovars L1 to L3. Recent studies reported on several pathogen and host genes related to disease severity and tissue tropism [5, 21–24], but these studies focused primarily on the molecular basis underlying the disparities between ocular, genital, and LGV associated genovars. Although we did not find any indication for tissue tropism within the urogenital strains, the possible role of tissue tropism on the prevalence of anorectal LGV infections could not be determined, as our study did not include urogenital samples from MSM. However, recent studies and case reports have described urethral and pharyngeal LGV infections in MSM, and urogenital and cervical infections among women [20, 25–27], suggesting at least no specific preference of LGV-inducing strains to solely infect anorectal tissue. Since anorectal LGV infections primarily occur in a subpopulation of HIV infected MSM with high risk sexual behaviour and high numbers of sexual partners, it is possible that this is also due to differences in host immunity, sexual behaviour and network associated factors. The lack of knowledge on the pathogenicity and transmission of LGV-inducing strains indicates the need for further research to clarify the predominant occurrence of anorectal LGV infections in comparison to urogenital and pharyngeal LGV infections.

To further investigate the possible role of tissue tropism in the distribution of *C. trachomatis* strains, we compared concurrent *C. trachomatis* strains detected in women at multiple anatomic locations. We did not observe any significant differences in the proportion of urogenital, anorectal or pharyngeal infections between clusters suggesting that *C. trachomatis* strains do not preferentially infect urogenital, anorectal, or pharyngeal tissue. Thus, these findings contradict previous studies reporting that the prevalence of *C. trachomatis* genovars varies by anatomical site and that genovar G is more commonly found in the anorectal tract [8, 10, 12, 13]. Our findings suggest that there is no strain that can be specifically associated with anorectal infections, as the anorectal *C. trachomatis* infecting strain is generally identical to strains causing a concurrent pharyngeal or urogenital *C. trachomatis* infection. We did identify some discordant infections among women with concurrent infections at multiple anatomic locations. Although these discordant infections may best be explained by sexual risk behaviour, causing women to concurrently get infected with multiple different *C. trachomatis* strains, we could not identify any significant difference in sexual risk behaviour characteristics compared to those with concordant infections. The most likely reason for discordant infection remains separate infections due to different sexual partners, as all women were STI clinic visitors who are known to be engaged in more risky sexual behaviour.

Some potential limitations of this study should be noted. We excluded a large number of samples as these were no longer available at the public health laboratory, had insufficient chlamydial DNA for typing, or could not be fully typed. Exclusion of these samples resulted in the inclusion of only a limited number of pharyngeal samples with the potential of a biased population. Another limitation is the difference in sample collection between high-risk and low-risk visitors of the STI outpatient clinic. Visitors were allocated to a standard or limited screening protocol depending on reported sexual behaviour. Criteria for a standard approach were: having STI related physical complaints; being notified of STI exposure by a sexual partner; having been paid for sexual contact in the past 6 months and for males, having had sex with men in the past 6 months [7, 16]. In the standard protocol, samples were taken by a trained nurse, and in the limited protocol these were self-swabs. Although women were carefully instructed on how to collect self-swabs, careless self-taken anorectal swabs by low-risk women might have been contaminated by a urogenital *C. trachomatis* infection due to the short anogenital distance.

## Conclusions

Using high-resolution multilocus sequence typing we found no indication for tissue tropism in urogenital *C. trachomatis* strains. Combined with results from previously conducted studies, we hypothesize that MSM and heterosexuals have different distributions of *C. trachomatis* strains due to their separate sexual networks.

Further research needs to provide more insight in the predominant occurrence of anorectal LGV infections in comparison to urogenital or pharyngeal LGV infections and whether or not this can be explained by tissue tropism.

## Authors’ original submitted files for images

Below are the links to the authors’ original submitted files for images. Authors’ original file for figure 1 Authors’ original file for figure 2 Authors’ original file for figure 3 Authors’ original file for figure 4
