# Supplementary figures and images for: No indication for tissue tropism in urogenital and anorectal Chlamydia trachomatis infections using high-resolution multilocus sequence typing
Source: BMC Infect Dis. 2014 Aug 26;14:464. doi: 10.1186/1471-2334-14-464 (PMC4155098; doi:10.1186/1471-2334-14-464)

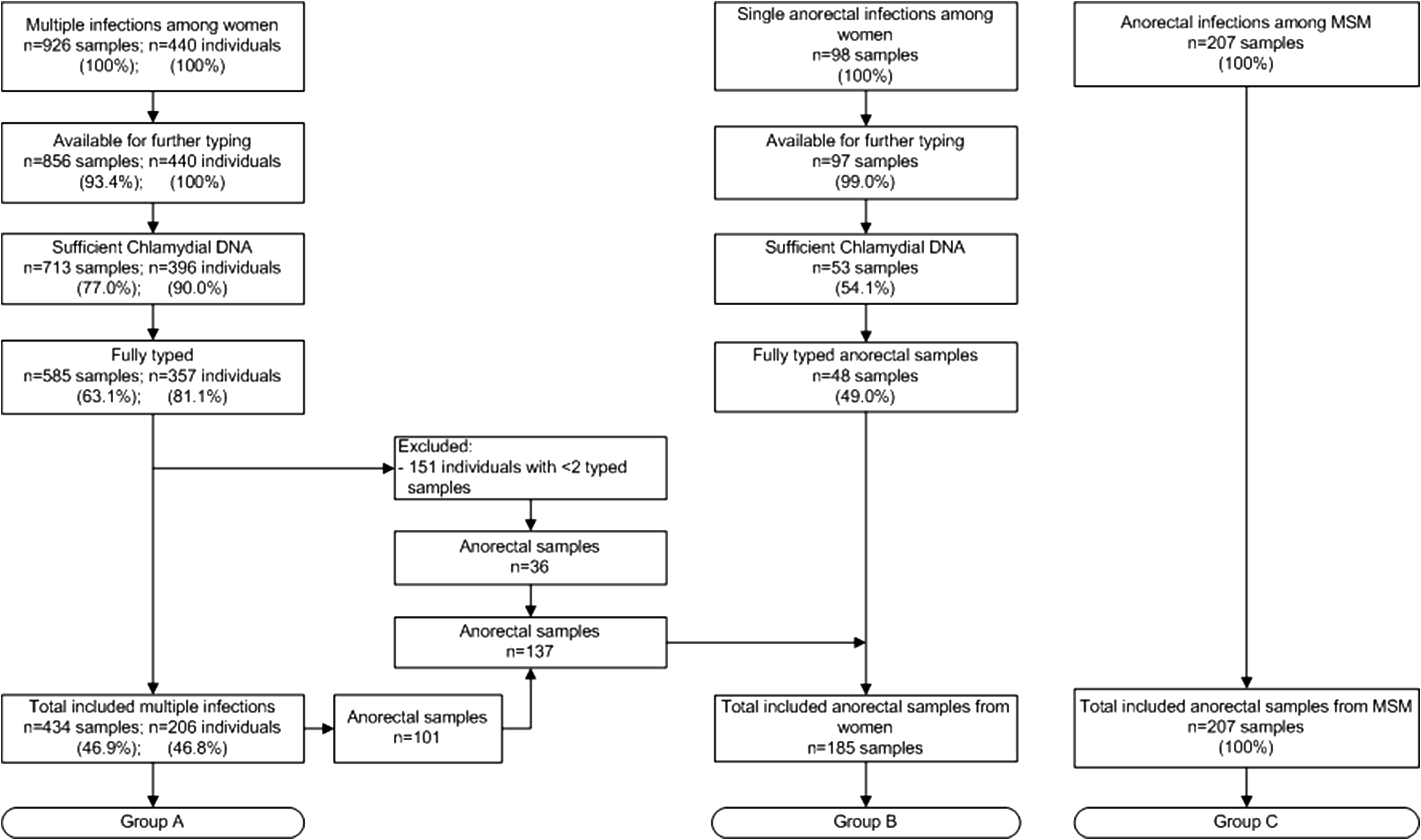

Supplement: Supplementary file 1 — Authors’ original file for figure 1 [file 12879_2014_3759_MOESM1_ESM.tiff]

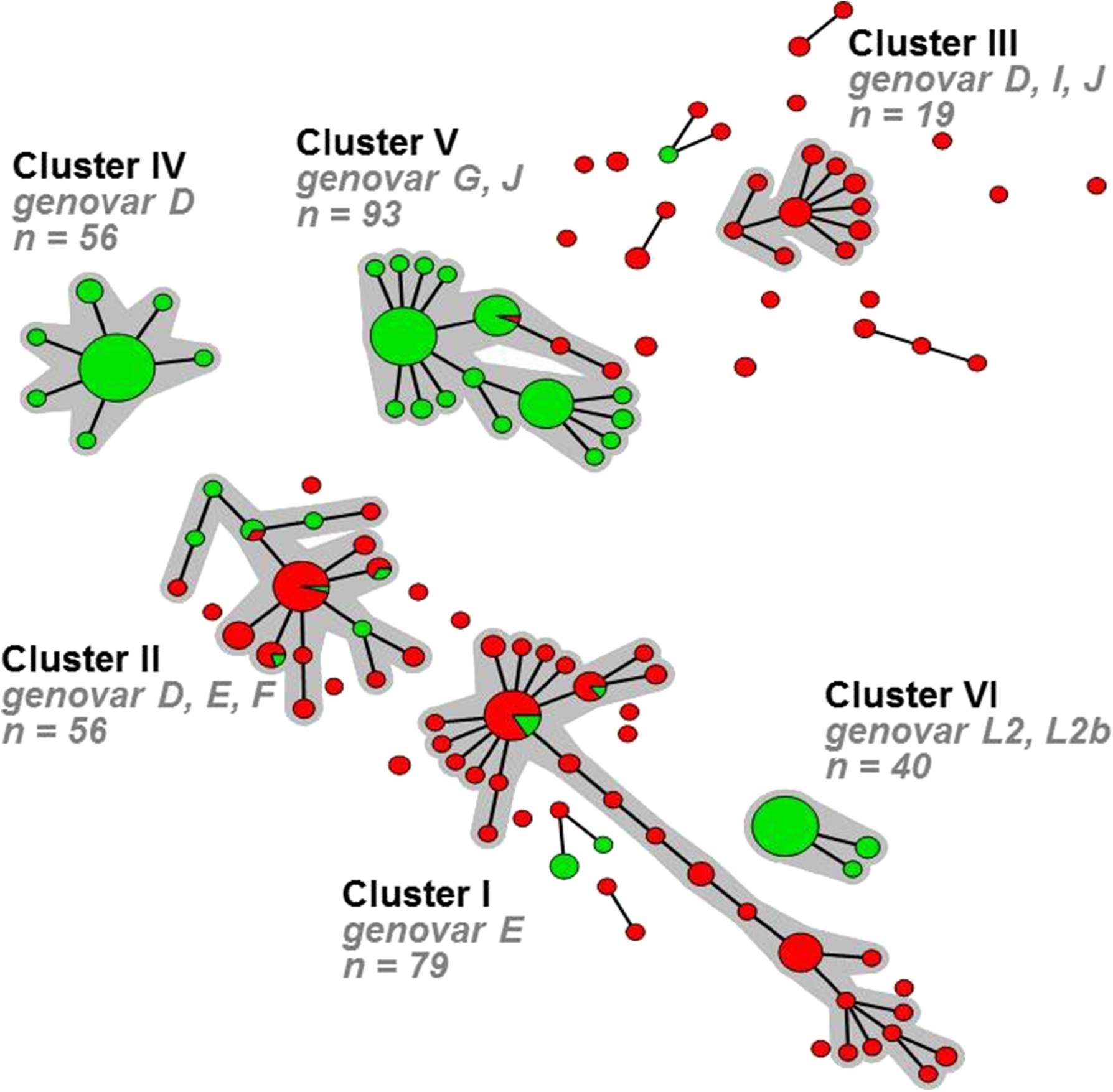

Supplement: Supplementary file 2 — Authors’ original file for figure 2 [file 12879_2014_3759_MOESM2_ESM.tif]

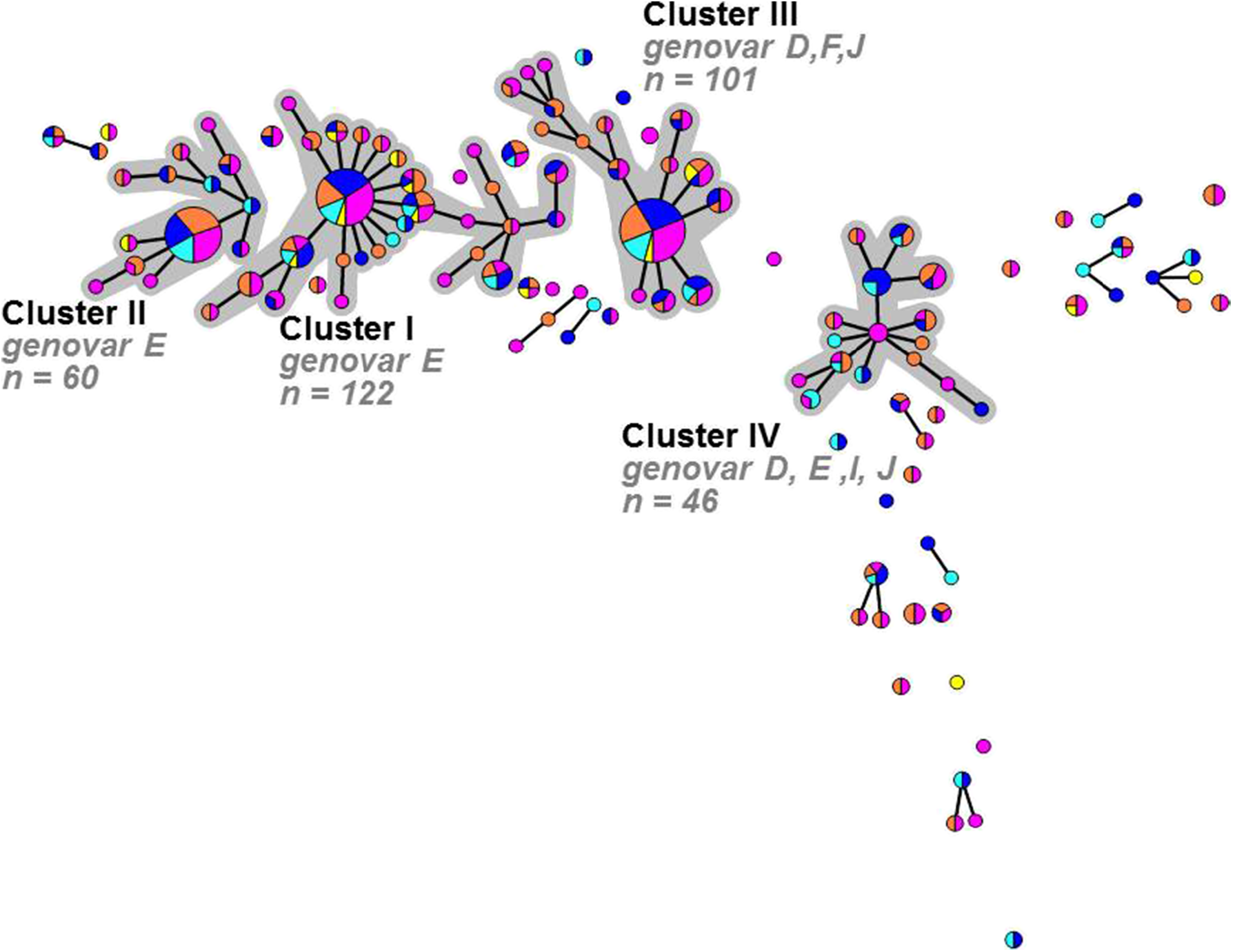

Supplement: Supplementary file 3 — Authors’ original file for figure 3 [file 12879_2014_3759_MOESM3_ESM.tif]

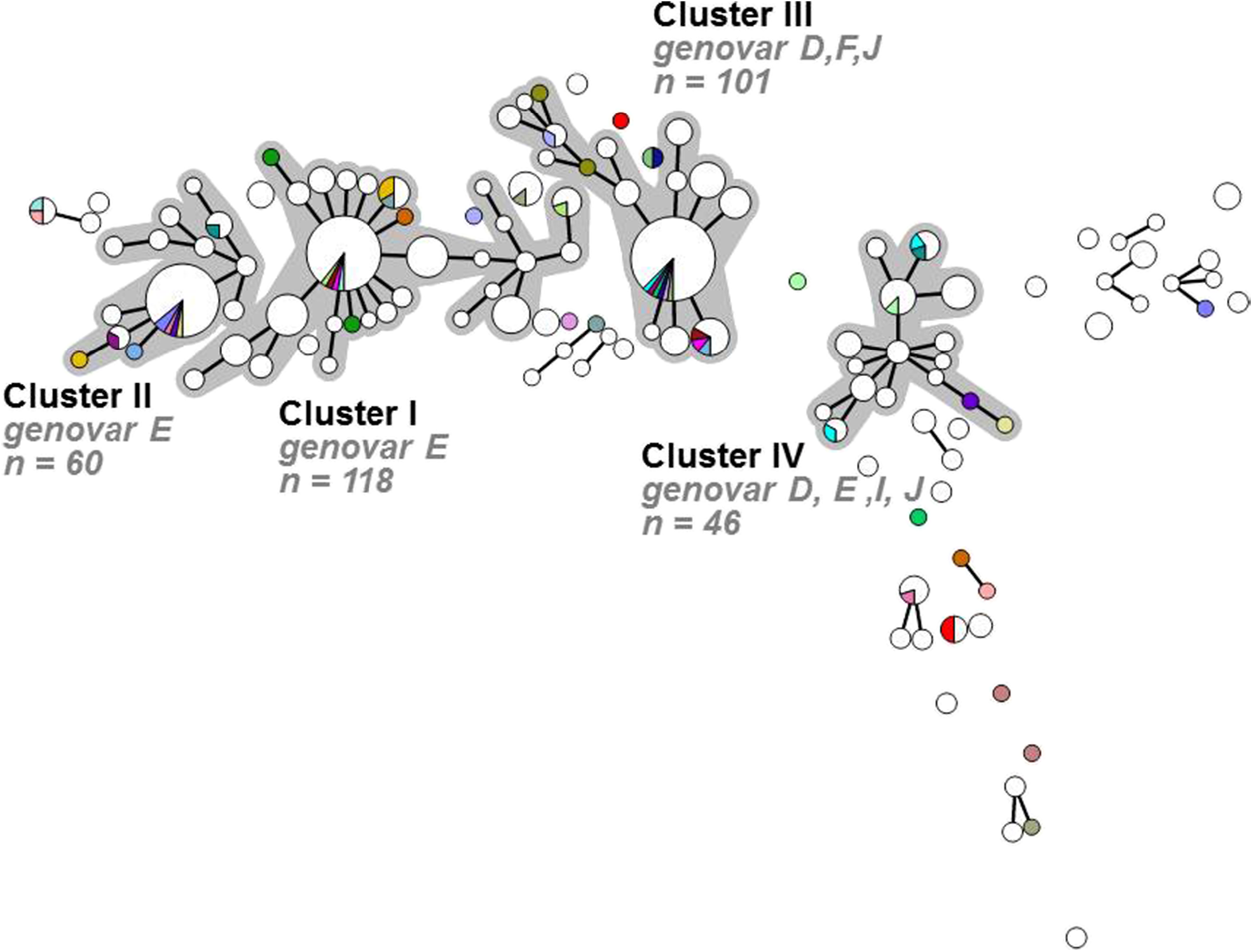

Supplement: Supplementary file 4 — Authors’ original file for figure 4 [file 12879_2014_3759_MOESM4_ESM.tif]
